# Seed-Mediated Synthesis of NiPt-Alloy-Tipped CdSe/CdS Nanocrystals for Photocatalysis

**DOI:** 10.1021/acs.chemmater.5c03047

**Published:** 2026-03-19

**Authors:** Mareike Dittmar, Julia Voß, Sebastian Hentschel, Lars Klemeyer, Dorota Koziej, Dennis Bonatz, Charlotte Ruhmlieb, Tobias Kipp, Alf Mews

**Affiliations:** † Institute of Physical Chemistry, 14915 University of Hamburg, Grindelallee 117, 20146 Hamburg, Germany; ‡ Institute for Nanostructure and Solid-State Physics, 14915 University of Hamburg, Luruper Chaussee 149, 22761 Hamburg, Germany

## Abstract

We present a method for preparing NiPt-alloy tips on CdSe-core/CdS-shell dot-in-rod nanoparticles (DRs). The formation of the NiPt tips is separated into a two-step synthesis, where first Ni tips are grown, which then serve as nucleation sites for Pt before an alloying process occurs. Thus, the NiPt tips are formed in a seed-mediated approach. We find that the reactivity of the Ni tips can be controlled by oxygen treatment, while the reactivity of the lateral surface of the DRs can be tuned by ligands. Monitoring the growth dynamics of the NiPt reveals that an oxide layer on the Ni tips delays the nucleation of Pt, but it does not inevitably prevent it if a combination of oleic acid and oleylamine initiates oxide-layer conversion due to oleate formation. Without conversion, the oxide layer can be utilized to inhibit the NiPt formation. The choice of ligands can be exploited to enable or prevent separate Pt particle growth on the lateral semiconductor rod surface. Especially the DRs with NiPt-alloy tips show superior activity toward HER in both electrocatalytic and photocatalytic experiments.

## Graphical abstract

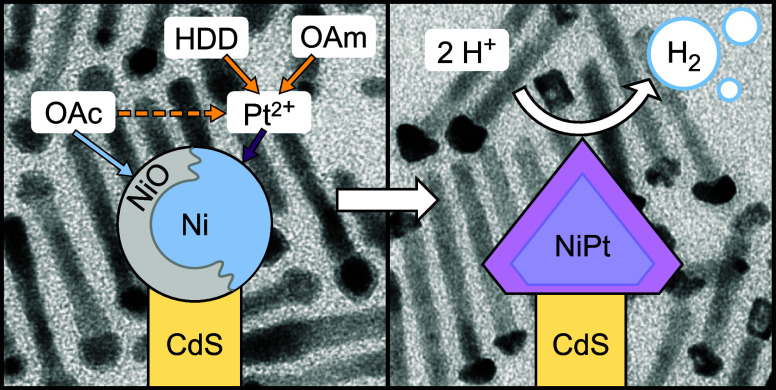

## Introduction

Energy storage is one of the biggest challenges of humankind. One way of storing energy is to convert solar energy into chemical energy, for example, by splitting water into hydrogen and oxygen. Both the hydrogen evolution reaction (HER) and oxygen evolution reaction (OER) can be realized electrochemically by using catalysts.

Metal nanoparticles are known for their applicability in electrocatalysis. In particular, Pt nanoparticles immobilized on an electrode are highly efficient at catalyzing the HER under acidic conditions.^1^ In alkaline media, it has been found that Ni and NiPt nanoparticles on electrodes efficiently catalyze the electrochemical HER.^2–5^ Generally, as compared to pure metal nanoparticles, alloyed metal nanostructures like NiPt offer the possibility of superior performances in various electrocatalytic applications, which is due to an altered electronic structure or modified surface reconstruction.^6–8^

Instead of using metal nanoparticles as an electrocatalyst on an electrode within an electrochemical cell, it is also possible to combine a metal nanoparticle with a semiconductor nanostructure in order to form a photocatalyst. Here, the semiconductor serves as the photon-absorbing entity from which charge carriers are transferred to the metal part with its catalytically active surface. Such semiconductor/metal heterostructured photocatalysts offer the possibility of directly converting solar energy to chemical energy.

It has been shown that elongated CdS-based nanoparticles, like CdS rods or CdSe-core/CdS-shell dot-in-a-rod nanostructures (DRs), equipped with either monometallic^9–12^ or bimetallic^13,14^ cocatalysts are promising HER photocatalysts. The reason for the suitability of CdS as the light-harvesting semiconductor species for a photocatalytic HER is its band structure, with the conduction band edge energy above the redox potential of the redox pair H^+^/H_2_.^15^ In CdSe/CdS DRs, an efficient photoexcited charge carrier separation is promoted, since the holes quickly localize in the CdSe-dot region, while electrons are intrinsically less localized in the semiconductor structure and thus can efficiently migrate to the metal.^9,16,17^

Such elongated semiconductor/metal nanoparticles can be fabricated using presynthesized rods or DRs as heteronucleation sites for the metal nanoparticles to form. Here, the nucleation can take place on the lateral surface of the rods or on their geometrically distinct tips.^18–21^ While the ability to control the position and quantity of metal sites on a semiconductor surface is desirable, it is not trivial to achieve. The number of metal nanoparticles per heteronanostructure has been shown to be relevant for the photocatalytic activity, with a single metal particle outperforming multiple.^22–24^

So far, a large variety of metals like Au,^10,13,25,26^ Pt,^9,13,16,23,27,28^ Ag,^14^ Ni,^11,29,30^ Pd,^12,14,26^ AgPd,^12^ AuPt,^13^ and AuPd,^14,26^ have been combined with rod-shaped CdS-based semiconductor nanostructures for HER. Pt-tipped CdS-based heterostructures demonstrate the highest photocatalytic HER yields in water under alkaline conditions.^31–33^ This contrasts with the aforementioned electrocatalysis experiments, which suggest that platinum exhibits higher catalytic activity in acidic water. However, acidic conditions are not conducive to photocatalysis with CdS-based rods because they damage the colloidal stability determined by the ligands. The reason for the comparatively high HER yield of Pt-CdS-based heteronanostructures in alkaline conditions is the result of an efficient hole scavenging.^31^

The high activity of Ni and NiPt nanoparticles demonstrated in electrochemical catalysis in alkaline solutions suggests their preferential use also in semiconductor/metal nanoparticle photocatalysts. Ni-decorated CdS-based rods have been reported, and photocatalytic experiments have been conducted.^11,29^ The synthesis of CdS rods with alloyed NiPt tips was achieved by the simultaneous reduction of the relevant precursors. Controlling the number of tips was challenging^34^ and no photocatalytic experiments have been shown. Instead of a simultaneous reduction of two precursors, alloys can be prepared by the subsequent growth of two different metals via a seed-mediated alloying process.^35,36^

In this work, we demonstrate a seed-mediated approach for preparing alloyed NiPt tips on CdSe/CdS DRs by the subsequent growth of Ni and Pt. Advantageously, in this approach, the Ni sites determine the position of the emerging NiPt nanoparticles: once Ni-tipped DRs are prepared, the tips can be transformed to NiPt. A thorough examination of the formation mechanism reveals that NiPt can be produced from Ni tips with and without an oxide layer. This allows Ni-tipped DRs to be handled under ambient conditions, for example, during cleaning. Analyzing aliquots taken from the synthesis after different reaction times reveals that an oxide layer delays only the alloying process. We show that the combination of oleic acid (OAc) and oleylamine (OAm) eliminates the oxide layer because of the formation of oleate through an acid–base reaction of OAc and OAm. The OAc is also shown to be relevant for controlling the position for Pt nucleation. Without OAc, Pt nucleates directly on the DR surface. Consequently, we show that by using either Ni tips with or without an oxide layer and by using or omitting OAc, it is possible to deliberately prepare NiPt-tipped DRs, NiPt-tipped DRs decorated with Pt particles on their lateral surface, or Ni-tipped DRs decorated with Pt dots. We discuss a model that shows how the transformation of the oxide layer, the reduction of Pt, and the alloying takes place. Finally, we show the first results of electrochemical and photocatalytic measurements to prove the activity of the NiPt-tipped CdSe/CdS DRs in the HER process.

## Results and Discussion

### Synthesis of NiPt Tips on CdSe/CdS DRs

Starting point for the NiPt-tip synthesis is CdSe/CdS DRs, presynthesized by a standard hot-injection process.^37^ For more information on the DR structures, see Supporting Information (SI). UV–vis absorption and fluorescence spectra, as well as fluorescence decay curves, are shown in Figure S2.

First, Ni was grown on the apexes of the CdSe/CdS DRs. This has been realized by a hot injection of CdSe/CdS DRs dispersed in trioctylphosphine into a solution of Ni acetylacetonate, oleic acid (OAc), oleylamine (OAm), and 1,2-hexadecanediol (HDD) in diphenyl ether under an inert atmosphere at 200 °C. Details are given in the Experimental Section. After their synthesis, in our standard procedure, the Ni-tipped DRs were handled under ambient conditions during the cleaning process and storage. Figure 1a shows a representative transmission electron microscopy (TEM) image of such prepared Ni-tipped DRs. The rod-shaped semiconductor part of these structures exhibits average dimensions of 32 ± 5 nm in length and 5.1 ± 0.6 nm in diameter. For about 50% of all structures, both apexes are decorated with Ni, while nearly all other structures exhibit only one Ni tip. The Ni tips appear predominantly spherical in the TEM images with an average diameter of 7.3 ± 0.9 nm. It is known that the (001̅) and (001) end facets of the DRs exhibit different reactivities.^37,38^ The (001̅) facet has the highest surface energy because of its S termination,^34^ leading to a preferential nucleation of metal atoms.^21^ However, at the opposite end of the DRs, there are also sulfur-rich higher-indexed facets,^20^ where metal nucleation can occur with a rather high preference, which then leads to DRs with two metal tips. The optical properties of the Ni-tipped DRs are shown in Figure S2 and discussed in the SI.

**Figure 1:**
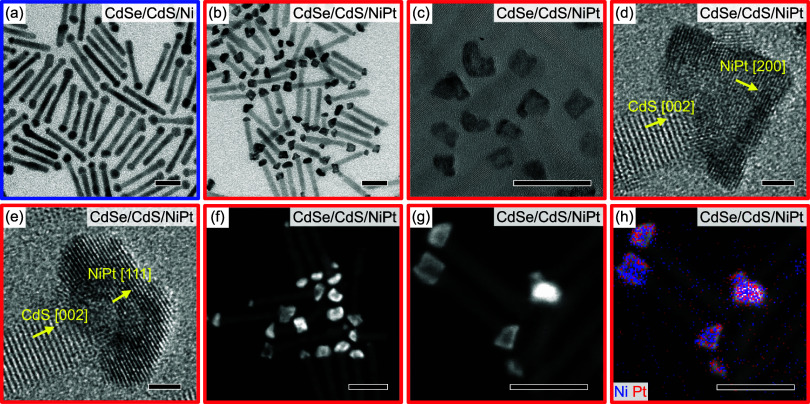
(a–c) TEM images of CdSe/CdS DRs with (a) Ni tips and (b,c) NiPt tips. Scale bars are 20 nm. (d,e) HRTEM images of NiPt tips with crystallographic directions as indicated. Scale bars are 2 nm. (f–h) HAADF-STEM images of DRs with NiPt tips. In panel (h), the image of panel (g) is overlaid by elemental EDX maps for Ni (blue) and Pt (red). Scale bars are 20 nm.

In order to turn the Ni tips into NiPt, Ni-tipped DRs were dispersed in a solution of Pt acetylacetonate in dichlorobenzene. This solution was injected into a hot solution of OAc, OAm, and HDD in diphenyl ether under an inert atmosphere at 200 °C. Details can be found in the Experimental Section. Figure 1b shows a representative TEM image of the emerging nanostructures obtained after a reaction time of 7 min for a direct comparison to the Ni-tipped DRs of Figure 1a. The tips in Figure 1b have a different contrast, are more angular, and slightly faceted compared to those in Figure 1a, indicating a structural change from pure Ni to NiPt tips. This is in line with reports on alloyed NiPt nanoparticles exhibiting pronounced faceting.^39,40^ Some NiPt tips appear to have a darker shell around a brighter core, as best seen in the higher-magnified TEM image in Figure 1c, indicating an inhomogeneous composition. The average NiPt-tip diameter, measured at the thickest cross-section perpendicular to the rod axis, is 8.0 ± 1.0 nm. The rod-shaped semiconductor part of these structures exhibit average dimensions of 30 ± 5 nm in length and 5.0 ± 0.6 nm in diameter, the same as before the Pt treatment. The fraction of structures with both apexes decorated with metal has slightly increased to about 60%. The similar number of DRs with two metal particles before and after Pt treatment indicates that Pt nucleation on the Ni tips is thermodynamically favored over nucleation at the DRs’ tipless sites. In rare cases, however, Pt nucleation also occurs opposite the NiPt tip. The optical properties of the NiPt-tipped DRs are shown in Figure S2.

Figure 1d,e shows high-resolution TEM (HRTEM) images of two exemplary NiPt tips attached to the respective DR. The visibility of the lattice planes proves the crystallinity of both the DR and the tip. The closed-packed stacking along the [002] direction of the hexagonal CdS is continued by the closed-packed stacking along the [111] direction of the cubic NiPt, as can be deduced from Figure 1e. The facets of the tip shown in Figure 1d are formed by (200) lattice planes. Details on the assignment of the NiPt lattice planes and the analysis of the lattice-plane spacings can be found in the SI. The spacings exhibit values between those of Ni and Pt, varying slightly from tip to tip. To gain more insights into the distribution of Pt within the NiPt tips, high-angular darkfield scanning TEM (HAADF-STEM) and energy-dispersive X-ray spectroscopy (EDX) measurements have been performed. Figure 1f shows a representative overview of the HAADF-STEM image. The tips typically exhibit a brighter, shell-like region surrounding a darker core. As the HAADF-STEM contrast is strongly dependent on the masses of the atoms in the sample, this indicates an increased amount of Pt near the surface of the tip. Interestingly, some DRs with distinct NiPt tips on one apex exhibit a smaller tip with high brightness on the other, indicating additional Pt nucleation during the last synthesis step. The comparison of the higher-resolution HAADF-STEM image in Figure 1g with elemental EDX maps for Ni and Pt (Figure 1h) reveals that the tip core is more Ni-rich, while the tip shell contains more Pt. EDX can also be used in TEM imaging to yield the overall composition of larger sample areas. EDX data corresponding to TEM images showing more than 1000 DRs revealed a Pt-to-Ni ratio of 50:50. Details on the analysis can be found in the SI. It is worth noting that the Pt-to-Ni ratio depends on the precursor amounts used. By increasing or decreasing the Pt amount in the injection solution, NiPt alloys with Pt contents between 45 and 70% were prepared, as measured by XRD. Further details are provided in the SI.

We want to compare our synthesis routes to previously reported routes for Ni-tipped DRs, alloyed NiPt nanoparticles, and NiPt-tipped DRs. Our route to synthesize Ni tips on DRs somehow resembles the route reported by Nakibli et al. in ref 11. The most important general differences from that synthesis are that we additionally employed OAc and used a different solvent. We found that this increases the reproducibility and simplifies the process. OAc has also been used by Ahrenstorf et al. in a coreduction reaction of Ni and Pt in order to achieve colloidal NiPt nanocrystals not attached to any semiconductor structures.^41^ Here, without any Pt precursor present, no formation of pure Ni particles has been reported. Since in our case, Ni tips are formed without any Pt precursor, this proves the role of DRs as nucleation sites for the Ni-nanoparticle formation. Our reaction conditions to alloy the Ni-tips on DRs by Pt resemble the ones reported by Habas et al. for the nucleation and growth of Pt (and NiPt) tips, emerging by (co)­reduction of Pt (and Ni) precursors.^34^ In contrast to the coreduction reaction, in our alloying reaction, we employ Ni-tipped DRs as precursor, making it possible that NiPt only forms at the Ni tips.

### Reaction Process of the NiPt-Tip Formation

In order to investigate the formation of the NiPt tips in more detail, aliquots of the reaction solution were taken after different reaction times *t*_r_ during NiPt-tip synthesis. Figure 2 shows representative TEM images of emerging products, corresponding to reaction times of between 1 and 5 min. Those tips that can be identified as NiPt based upon their shape and contrast are marked by red circles. Obviously, the transformation from pure Ni to NiPt tips takes place only after a certain reaction time. The conversion only started after *t*_r_ ≈ 2 min; the vast majority of tips were converted after *t*_r_ ≈ 5 min. From TEM images, the tip size of the particles in each aliquot was determined. Plotting the results vs reaction time (see Figure S5a), no significant change in the tip size was determined for *t*_r_ ≤ 4 min. The tip size appeared to increase slightly at a reaction time of 5 min. This is consistent with the observed time frame for the majority of Ni tips to convert to NiPt.

**Figure 2:**
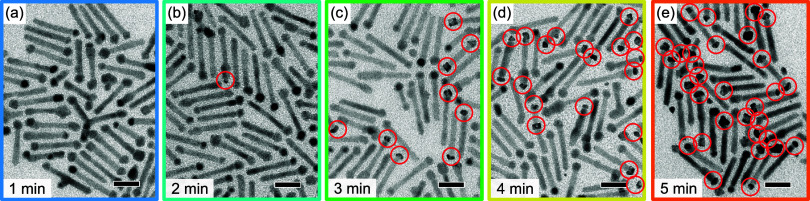
(a–e) TEM images of the aliquots taken at different reaction times during NiPt-tip formation. Scale bars are 20 nm. The tips marked by red circles have been assigned to NiPt on the basis of their shape and contrast.

Figure 3a shows powder X-ray diffraction (PXRD) data of the Ni-tip/DR sample (cf. Figure 1a), the aliquots corresponding to reaction times between 1 and 5 min (cf. Figure 2), and the NiPt-tipped DRs after 7 min reaction time (cf. Figure 1b–h). Thus, the entire reaction process is represented. Additionally, reference data of Pt (top) and CdS and Ni (bottom) are given. For *t*_r_ ≤ 2 min, no change of the PXRD data is evident. The diffractograms are largely dominated by the CdS signal, superimposing the expected Ni reflections at approximately 44.5° and 52.0°. After *t*_r_ = 3 min, a reflection at around 41.5° arises, which develops to a very prominent signal with progressing reaction time. This peak, positioned between the (111) reflections of Ni and Pt, represents the (111) reflection of NiPt with a Pt content of about 61%, as deduced from Vegard’s law.^42^ This value is higher as compared to the value of 50% obtained from the ensemble EDX measurements (see above and the SI). This confirms the presence of unalloyed nickel in the core, despite the dominance of the NiPt reflection at *t*_r_ = 7 min. This is because the NiPt shell occupies a larger volume than the core within the structure.

**Figure 3:**
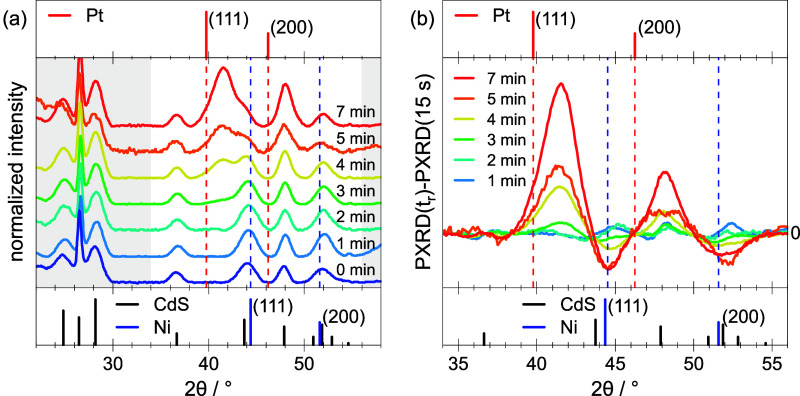
(a) PXRD data of samples belonging to reaction times from *t*_r_ = 0 min (before Pt treatment) to *t*_r_ = 7 min (final product). The data have been normalized to the (102) CdS reflection at 2θ ≈ 37° and vertically shifted for clarity. Top and bottom subpanels give literature data for Pt (PDF#: 00-004-0802), Ni (PDF#: 00-004-0850), and CdS (PDF#: 00-041-1049). (b) Difference between normalized diffractograms shown in panel (a) and the normalized diffractogram measured for *t*_r_ = 15 s.

To better illustrate the development of Ni and NiPt reflections over the course of the reaction, the diffraction patterns shown in panel (a) have been adjusted by subtracting a reference diffraction pattern. This reference was measured for an aliquot taken from the reaction solution shortly after precursor injection (*t*_r_ = 15 s). Figure 3b shows the processed diffraction data in the relevant diffraction-angle range. Here, again, the development of the (111) NiPt reflection for reaction times larger than 3 min is clearly visible. Additionally, the evolution of a reflection at about 48.0° is now observable. This reflection can be assigned to the (200) reflection of NiPt, for which Vegard’s law delivers a similar Pt content as given above. Besides increasing diffraction signals, also a decrease in signal with increasing reaction time can be observed at about 44.5° and 52.0°. This shows that the content of pure Ni decreases while the NiPt alloy evolves.

For a TEM investigation of the transformation from Ni to NiPt, the aliquot taken after *t*_r_ = 3 min was chosen, since here the original Ni tips and alloyed NiPt coexist. Figure 4a shows an HRTEM image where the two different species are recognizable: faceted NiPt tips and rather spherical Ni tips. The spherical tip marked with a circle clearly shows a core/shell structure. Such a core/shell structure can be recognized in almost every spherical tip in the image. On very close inspection, similar shells can also be observed in Ni-tipped DRs before the alloying reaction (cf. Figure 1a). This shell is assigned to oxidized Ni.

**Figure 4:**
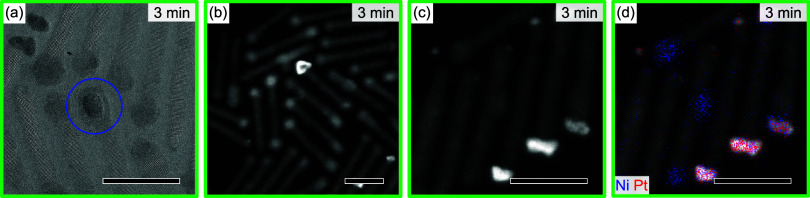
Aliquot at 3 min reaction time during NiPt tip formation. (a) HRTEM image, (b) HAADF-STEM image, (c) HAADF-STEM image, and (d) EDX maps for Ni (blue) and Pt (red) superimposed on the image of (c). Scale bars are 20 nm.

Figure 4b shows an HAADF-STEM image of the same sample. The different tip types exhibit different levels of brightness. Spherical tips are less bright; in some of them, a darker shell can be resolved, indicating the Ni/oxidized-Ni core/shell structure. The faceted NiPt tips are brighter because of the higher atomic number of Pt as compared to Ni and its oxide layer. Figure 4c,d compares an HAADF-STEM image with elemental EDX maps for Ni and Pt. It can be seen that the larger spherical tips contain only Ni, while the faceted tip contains Ni and Pt, probably as an alloy. Interestingly, some small spherical particles of pure Pt can be recognized that additionally nucleated on the DR surfaces, mainly on the DR’s unoccupied tip.

In our TEM investigation, no intermediate tip structure that represents the transition from spherical Ni tips to faceted NiPt tips could be observed. Such an intermediate structure could, e.g., consist of small Pt seeds attached to a Ni or oxidized-Ni surface. This suggests that the actual transformation of an individual tip occurs on short time scale, which cannot be resolved in our experiment. The point in time for this transformation, however, is different for each tipped DR within the sample. It takes several minutes of reaction time to transform all of the tipped DRs of a sample. Based on the observations presented above, we assume that a delay in NiPt formation is caused by an oxidized layer on the Ni tips.

For further investigation, the synthesis of Ni tips on DRs, their processing into NiPt tips, and all cleaning and storage processes have been repeated under inert conditions to exclude the influence of oxygen or water. Again, a series of aliquots were taken for different reaction times. Figure 5 shows TEM images of such prepared Ni-tipped DRs (panel a) and of the aliquots corresponding to reaction times *t*_r_ of 15 s and 3 min (panels b and c, respectively). Similar to Figure 2, in Figure 5b, those tips are marked by red circles that could be identified as NiPt. Already after 15 s, about half of the Ni-tips were transformed to NiPt. For *t*_r_ = 3 min, all tips visible have transformed. This proves that NiPt formation is indeed drastically faster when the formation of an oxide layer at the Ni-tip surface is suppressed. The diameter of tips increased after *t*_r_ = 1.5 min (see Figure S5b), fully inline with the conversion of most of the Ni tips to NiPt.

**Figure 5:**
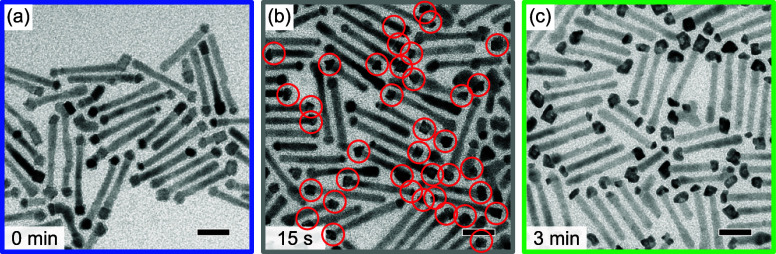
(a) TEM image of Ni-tipped DRs synthesized, cleaned, and stored under inert atmosphere. (b,c) TEM images of aliquots taken at different reaction times during NiPt-formation using the Ni-tipped DRs of panel (a) as precursor. The tips marked by red circles in panel (b) have been assigned to NiPt. Scale bars are 20 nm.

### Oxide-Layer Characterization and Conversion

Results so far suggest that the oxide layer, which is inherently formed in our standard procedure with Ni-tipped DRs handled under ambient conditions, is transformed or removed before the seed-mediated alloying with Pt begins. To characterize the oxide layer in more detail, high-energy-resolution fluorescence-detected X-ray absorption near-edge structure (HERFD-XANES) measurements were performed. Figure 6a shows normalized Ni K_α_ HERFD X-ray absorption spectroscopy (HERFD-XAS) data obtained for two Ni-tipped DR samples, as well as for macroscopic Ni and NiO reference samples. The Ni-tipped DR samples originated from the same batch, which was synthesized and processed under inert conditions. The sample solution was divided into two parts: one was kept under a nitrogen atmosphere, and the other was deliberately exposed to air.

**Figure 6:**
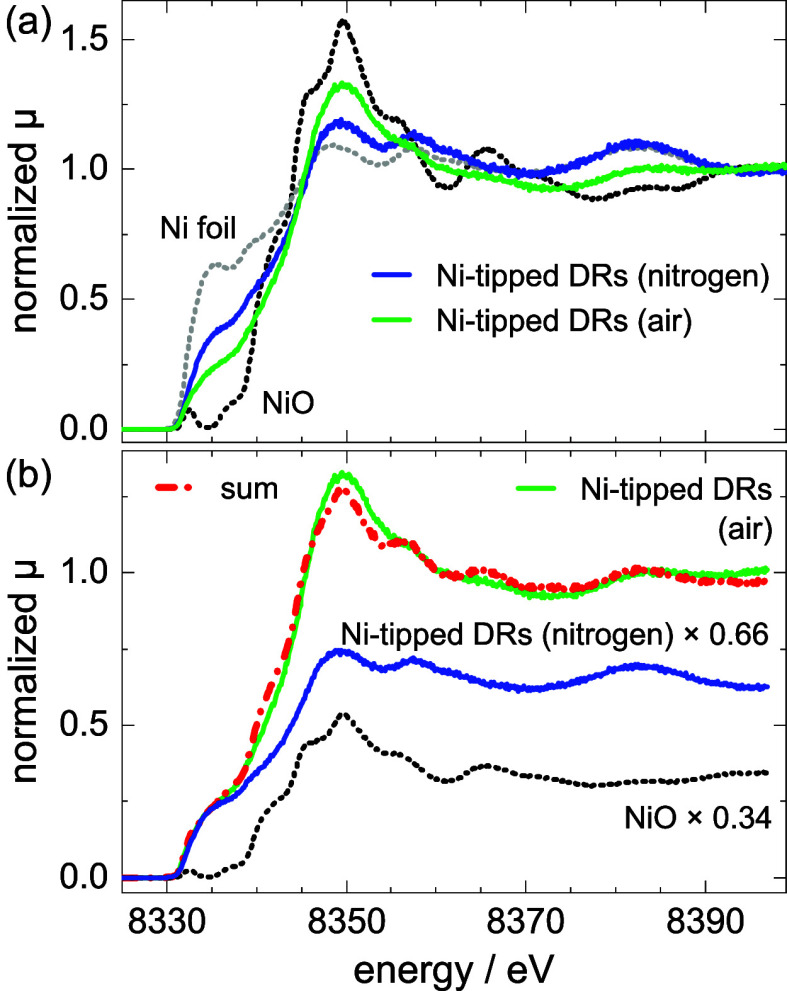
(a) Normalized Ni K_α_ HERFD-XANES spectra for the Ni-tipped DRs, handled under a nitrogen atmosphere (blue line) and exposed to air (green line), and reference spectra for Ni (Ni foil) and NiO. (b) Weighted normalized HERFD-XANES spectra of Ni-tipped DRs stored under nitrogen (blue line) and NiO (dotted black line) lead to a sum spectrum (dash–dotted red line) that fits well the normalized spectrum of Ni-tipped DRs that were exposed to air (green line).

The spectra of the Ni-tipped DRs differ in the positions and intensity ratio of the white line and the pre-edge feature, as can be seen in Figure 6a. None of the spectra matches a single reference spectrum. However, the spectrum of the Ni-tipped DRs that were exposed to air can be reproduced by superimposing the spectra of the sample stored under nitrogen conditions and the NiO reference. This is visualized in Figure 6b, in which the latter spectra are shown weighted by factors of 0.66 and 0.34, respectively, leading to a sum spectrum (dashed–dotted line) that coincides well with the spectrum of the sample exposed to air. This coincidence leads to the conclusion that the oxidized Ni shell in air-treated samples consists mainly of NiO rather than Ni­(OH)_2_, NiOOH, or other nickel–oxygen compounds. Further details on the HERFD-XANES analysis can be found in the SI.

Electron-energy loss spectroscopy (EELS) was used to investigate the oxidation state of Ni during our standard procedure for NiPt formation. Comparing EELS data of the aliquots belonging to *t*_r_ = 3 min and *t*_r_ = 7 min reveals that Ni^2+^ disappears over the course of the reaction and the Ni^0^ content increases (see Figure S8 in Supporting Information). Reoxidation of the Ni in the NiPt is inhibited by the alloying with Pt.^43^

To investigate the reasons for the oxide-layer transformation, the relevance of oleylamine (OAm), oleic acid (OAc), and 1,2-hexadecanediol (HDD) during NiPt-alloy formation is tested. In the experiment, Ni-tipped DRs with and without oxide layer were used as the precursor (see Figure S9 for TEM images). Figure 7 shows TEM images of the products of several syntheses (*t*_t_ = 7 min), each obtained with a similar ratio of Ni-tipped DRs and a Pt precursor.

**Figure 7:**
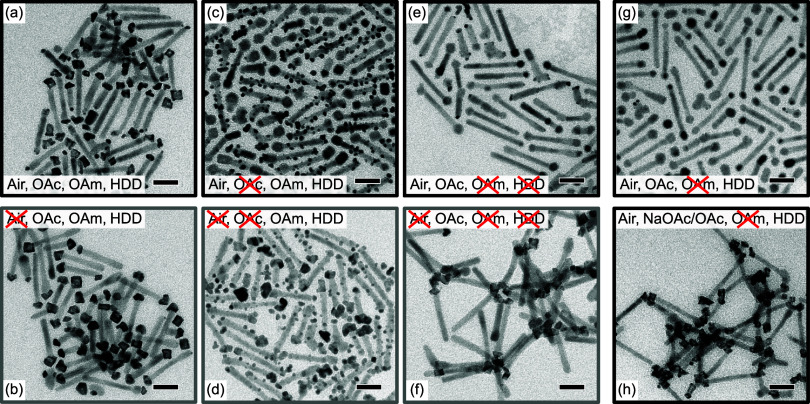
TEM images of the products obtained in syntheses using various reactants: (a) Standard synthesis using Ni tips handled under ambient conditions. (b) Same as panel (a) but using Ni tips handled under inert conditions. (c,d) Omitting OAc, using Ni tips handled under (c) ambient and (d) inert conditions. (e,f) Omitting OAm and HDD, using Ni tips handled under (e) ambient and (f) inert conditions. (g) Omitting OAm, using Ni tips handled under ambient conditions. (h) Omitting OAm and replacing half of OAc by NaOAc, using Ni tips handled under ambient conditions. All scale bars are 20 nm.

Figure 7a,b compares particles produced with precursor-Ni tips with and without an oxidized surface, respectively. In the latter case, the NiPt tips are slightly larger and even less side-nucleation of Pt on the DR surface occurred. The lower amount of side-nucleation hints at fewer surface defects that act as additional nucleation sites for Pt and whose formation is suppressed when air exposure is prevented.^44^

Figure 7c,d shows the corresponding particles that emerged from reactions in which OAc was omitted. For the Ni-tipped DRs handled in ambient conditions (panel c), the tips seem unchanged and there is no evidence for a NiPt formation, while Pt dots are distributed over the entire lateral surface of the DR. For the Ni-tipped DRs handled under exclusion of air (panel d), the tips were transformed into NiPt tips, and similar Pt dots occurred on the CdS surface. This behavior, on the one hand, proves that OAc plays an important role in the removal of the oxide layer in the standard synthesis. On the other hand, it suggests that OAc acts as a protecting ligand on the CdS surface. The additional nucleation of Pt occurs possibly at unprotected surface-defect sites on the CdS caused by the lack of OAc. OAc can in principle bind to CdS as an X-type ligand.^45^

The TEM images shown in Figure 7a–d clearly demonstrate that the choice of ligands is not only crucial for the formation of alloy metal tips but also essential to control the side-nucleation reaction of Pt particles on the semiconductor surface. Obviously, by using either Ni-tipped DRs under ambient or inert conditions and by using or omitting OAc, one can deliberately synthesize either only NiPt-tipped DRs (Figure 7a,b), Ni-tipped DRs with Pt dots on the DR lateral surface (Figure 7c), or NiPt-tipped DRs with Pt dots on the DR lateral surface (Figure 7d). PXRD measurements of the corresponding samples perfectly support these findings, showing reflections of either NiPt, Pt (with Ni masked by CdS), or NiPt and Pt, respectively (see Figure S10).

Figure 7e,f shows particles that emerged in reactions without the OAm and HDD. For the Ni-tips with an oxide layer (panel e), again no NiPt formation is observed. For precursor structures without oxide layer (panel f), however, most of the tips appear to be faceted, indicating the transformation from Ni to NiPt. Those tips tend to stick together. These observations show that also OAm or HDD play a significant role for the removal of the oxide layer in the standard synthesis. It can also be deduced that OAm or HDD acts as a ligand for NiPt that prevents agglomeration in the standard process. OAm has been reported to be a good ligand for Pt.^46^

Figure 7g shows particles that were formed in a synthesis with air-treated Ni tips, in which OAm was omitted, but HDD was still used. Since the results are very similar to the ones shown in panel e, this proves that HDD does not significantly influence the removal of the oxide layer. HDD is a reducing agent often used for metal ions.^46–49^

The previous results prove that both OAc and OAm are necessary for the conversion of the oxide layer of Ni tips treated under ambient conditions prior to the seed-mediated NiPt formation. Two processes are conceivable when OAc and the OAm are added together: On the one hand, amide formation; and on the other hand, an acid–base reaction. The amide formation is an equilibrium reaction in which water is formed. In our reaction, without water removal, water would tend to shift the equilibrium to the side of the reactants, and only small amounts of the amide should be present. In the acid–base reaction, OAc^–^ and OAm^+^ should form. To mimic an acid–base reaction, a control experiment has been performed in which half of the original amount of OAc was substituted by NaOAc, and the original amount of OAm was omitted. Figure 7h shows the emerging particles when Ni-tips with an oxide layer were employed as a precursor. Besides the fact that the tips of the particles stick together due to the lack of an OAm as ligand (cf. Figure 7f), it can be seen that the Ni tips transformed to NiPt, proving that OAc^–^ is responsible for the conversion of the oxide layer.

For the specific OAc^–^-induced conversion mechanism, one might expect etching processes or reduction of Ni cations within the NiO layer. A detailed TEM analysis of the tip diameter of the aliquots revealed no significant decrease under standard reaction conditions (see Figure S5). HRTEM and STEM investigations of the aliquot at 3 min reaction time (cf. Figure 4) revealed oxide-layer thicknesses around 1.8 nm (±0.3 nm). An etching of a layer thickness of more than 0.5 nm, i.e., a systematic decrease in tip diameter by more than 1 nm, should have been resolvable in the TEM analysis. Hence, we conclude that etching processes only play a subordinate role, while Ni^2+^ reduction within the NiO layer, which is a mechanism that conserves the tip diameter, is dominant. Here, OAc^–^ acts as a reducing agent for Ni cations.

### Pt Reduction and Seed-Mediated Alloy Formation

After conversion of the oxide layer, Pt can deposit onto the Ni tips. The prerequisite for NiPt formation is the reduction of Pt cations. This can occur via reducing agents in solution, such as OAm, HDD, and OAc. It is difficult to determine whether the reduction process takes place close to the particles in solution, with the Pt^0^ atoms subsequently attaching to the Ni, or whether the reduction occurs directly on the Ni-tip surface. A reduction of Pt cations could also take place in a galvanic exchange process owing to the potential differences between Ni and Pt, whereby Ni would directly reduce Pt^2+^ while dissolving as Ni^2+^.^50,51^ In an organic solvent, an anionic/X-type ligand would be required to stabilize the oxidized cation.^52^ Here, OAc/OAc^–^ plays this role. Since much less Pt or NiPt can be recognized in the absence of OAm and HDD (cf. Figure 7e,f) than in their presence (cf. Figure 7a–d), we conclude that reduction via OAm and HDD as reducing agents is dominant. The NiPt tips that form in the absence of the OAm and HDD (see Figure 1f) are smaller and less distinct than those formed with both reducing agents present (see Figure 1d). It is unclear whether, here, the necessary Pt cation reduction occurs via the weak reducing agent OAc or via galvanic exchange. Even the detection of Ni cations in the reaction solution would not be a conclusive proof of galvanic exchange, as they could also be produced by slight corrosion of the Ni tips under the prevailing reaction conditions.

The nucleation of reduced Pt^0^ at the Ni tip but also a galvanic exchange process from Ni to Pt would lead to the formation of a Pt shell around the original Ni tip. Although some NiPt tips show a core/shell-like structure in HAADF-STEM images, the PXRD data clearly demonstrate alloying of Ni and Pt. Consequently, an alloying process by interdiffusion of reduced and nucleated Pt atoms into the original Ni tips and of Ni atoms from the original tips into the new Pt-rich shells has to occur. This seed-mediated alloying process is accompanied by a transformation from Ni to faceted NiPt tips.

### Conclusion of the NiPt-Tip Formation

Our main findings concerning the synthesis of NiPt-tipped DRs are sketched in Figure 8. In order to transform Ni tips to NiPt, a Ni oxide layer, which is present in the circumstance of a synthesis under ambient conditions, has to be converted or removed (panel a). We assume that OAc^–^, originating from an acid–base reaction of OAc and OAm, acts as a reducing agent for Ni^2+^. Without OAc or OAm, the oxide layer is not converted. Ni without an oxide layer can transform to NiPt. For that, Pt^2+^ has to be reduced, emerging Pt^0^ has to nucleate on the Ni tip and diffusion of Pt into the Ni tip has to take place (panel b). OAc, OAm, and HDD each are reducing agents. OAm and HDD reduce Pt cations more efficiently than OAc. OAc has an additional function in the synthesis; it acts as a ligand on the lateral surface of the CdS DRs, which effectively hinders the side nucleation of Pt that would occur without passivation by OAc (panel c). By using Ni-tipped DRs with or without an oxide layer and by adding or omitting OAc, it is possible to set whether Ni- or NiPt-tipped DRs are created with or without Pt dots on the DR lateral surface.

**Figure 8:**
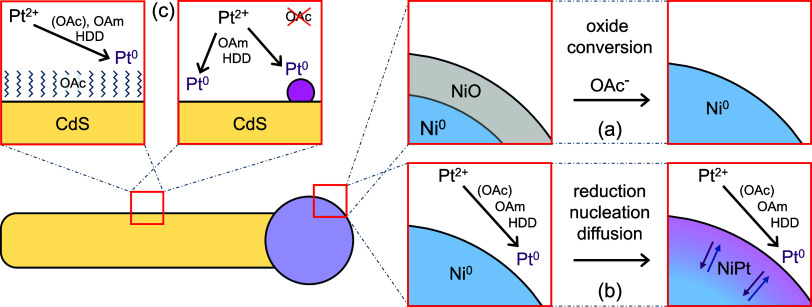
Scheme of the main findings concerning NiPt formation. (a) Ni oxide conversion to Ni^0^. (b) Reduction of Pt^2+^, nucleation of Pt^0^ and diffusion of Pt into the Ni. (c) Pt nucleation on the lateral surface of DRs guided by the presence or absence of OAc.

### Catalysis

To investigate the catalytic activity toward HER for NiPt-tipped CdSe/CdS DRs, both electro- and photocatalytic measurements were performed.

Electrocatalysis experiments are important to characterize, particularly, the metal part of the hybrid photocatalysts. They can be used to determine the overpotential required to drive a reaction on a catalyst’s surface, which is represented by the potential at which the catalytic reaction starts. The overpotential is an important parameter directly linked to the applicability of nanostructures in photocatalysis.

In Figure 9a, cathodic polarization curves of cyclic voltammetry (CV) measurements in 0.1 M KOH electrolyte at pH 13 are shown for differently tipped DR nanostructures immobilized on FTO electrodes. Besides the NiPt-tipped DRs, also DRs with pure Pt tips, pure Ni tips, and no metal tips were measured. Details on the different nanostructures are given in the SI. As can be seen, the overpotential is lowest for NiPt, then increases for Pt, and increases further still for Ni tips. This proves that NiPt is a promising candidate for HER catalysis in alkaline media.

**Figure 9:**
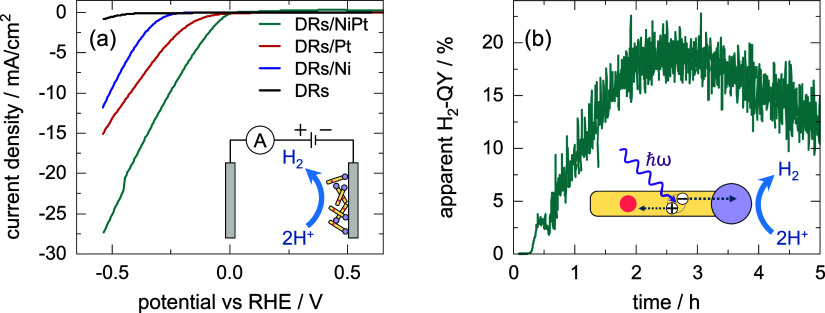
(a) Cathodic polarization curve in the potential range up to −0.55 V vs RHE of DRs as well as DRs with Pt tips, Ni tips, and NiPt tips. The scheme shows a typical experimental setup for electrochemical HER with immobilized DRs on an electrode. (b) Apparent H_2_-QY with an LED with a wavelength of 365 nm of NiPt-tipped DRs. The scheme shows the photon absorption, charge carrier separation, and photocatalytic HER in a metal-tipped DR.

Regarding photocatalysis, it has been previously reported that CdS-based rod-like structures with metal tips exhibited the highest H_2_ quantum yields (QYs) for very high pH values up to 14.7. This has been explained by a hole-shuttle mechanism, whereby OH^–^ ions efficiently extract holes from the semiconductor part of the photocatalysts. The emerging OH^·^ radicals then oxidize alcohols present in the solution, which were intended to act as the actual hole scavengers in experiments at lower pH.^29,31^ In later experiments, it has been shown that the OH^·^ radicals affect the stability of the catalysts by first decomposing the ligands and then the semiconductor.^33^ The application of photocatalysts in less harsh media would be desirable. However, to ensure comparability, here, we conducted photocatalytic experiments under conditions comparable to those reported in previous studies. More precisely, the conditions were set similar to those in ref 33. A mixture of 5 M KOH solution at pH 14.7 with 10% ethanol was used as the catalysis medium. Photoexcitation occurred with an LED at a 365 nm wavelength with a power density of 65 mW/cm^2^.

The photocatalyst employed was NiPt-tipped DRs, prepared using the standard procedure described above. The semiconductor DRs consisted of CdSe dots with a diameter of 2.3 nm encapsulated in a CdS shell, on average about 5.7 nm in width and 60 nm in length. The NiPt tips exhibited an average diameter of 10 ± 1 nm (for a TEM image, see Figure S11a). After their synthesis, the NiPt-tipped DRs were transferred to an aqueous solution via ligand exchange with mercaptoundecanoic acid (11-MUA). After precipitation, the NiPt-tipped DRs were redispersed in the alkaline catalysis medium, adjusting the concentration so that the optical density of the solution at the excitation wavelength of 365 nm was similar to that reported in ref 33.

Figure 9b shows the time evolution of the apparent H_2_-QY during the first 5 h of photocatalysis. The QY increases over time, reaching a maximum after about 2.5 h, before it decreases again. A similar behavior has been observed and thoroughly analyzed before in ref 33 for similar CdSe/CdS DRs equipped not with NiPt tips but with smaller pure-Pt tips. It was shown that the increase in the catalytic activity during the first hours of the reaction can be attributed to partial removal or thinning of an initially dense ligand shell around the hybrid nanostructures. This removal first has the positive effect of improving the accessibility of the nanocatalyst surface, which leads to a higher catalytic reactivity. With ongoing degradation of the ligand shell, however, aggregation of the particles takes place, followed by irreversible surface reactions, such as photocorrosion. Both effects can decrease the catalytic activity of the nanostructures.^33^

The maximum apparent QY of about 20% for our NiPt-tipped DRs (see Figure 9b) is nearly the same as for similar DRs equipped with smaller Pt tips (18%, see ref 33). An exact comparison of the QYs for different photocatalysts is difficult because the numbers strongly depend on multiple parameters, like the concentration of active nanostructures, their geometry, their structural integrity after phase transfer, or the density of ligands on their surface. It is worth noting that the fraction of DRs having no metal tip increased from 1.5% before ligand exchange to about 50% for the photocatalysis solution before the actual photocatalysis experiment and after additional cleaning (see SI, in particular Figure S11b). Since DRs without tips and NiPt-tips without DRs both contribute to the light absorption but do not significantly contribute to the photocatalysis, the actual QY of NiPt-tipped DRs might be significantly higher. Furthermore, since the apparent QY relates the H_2_ production to the number of absorbed photons, its value is affected by the size of the metal tips. Larger tips exhibit stronger absorption.^18,30^ Hence, decreasing the size of the NiPt tips might further increase the apparent QY. An in-depth discussion of the photocatalytic properties of NiPt-tipped CdS-based DRs, including effects depending on tip size and faceting and possibilities to increase the long-term stability by changing to a catalytic medium less harsh than pH 14.7, is beyond the scope of this work. Our first results demonstrate the high potential of NiPt-tipped DRs as efficient photocatalysts.

## Summary

We demonstrated a seed-mediated synthesis of alloyed NiPt metal particles on the tips of CdSe/CdS DRs by a subsequent growth of Ni and Pt. The NiPt tips exhibit a faceted surface and typically a core–shell structure, arising from a Ni-rich core and a Pt-rich alloyed shell.

The analysis of aliquots taken at different reaction times during the NiPt synthesis revealed that, in the standard procedure in ambient conditions, the Ni tips exhibit a NiO surface layer that delays the NiPt formation. This oxide layer is converted to a Ni^0^ surface over the course of the reaction. We found that OAc is key in the conversion of the oxide layer. For that OAc is deprotonated by OAm in an acid–base reaction to form OAc^–^, which serves as a reduction agent for NiO. After obtaining a Ni^0^ surface on the Ni tips, the reduction of Pt takes place mainly by the reducing agents OAm and HDD. An interdiffusion leads to the alloying of Ni and Pt after Pt nucleation. We found that OAc is also essential for controlling Pt nucleation on the lateral surfaces of the DRs. Since it functions as a ligand, its absence in the reaction solution leads to the pronounced formation of Pt nanoparticles on the semiconductor surface. The multiple functions of OAc, combined with the presence or absence of an oxide layer, allow for a controlled synthesis of either NiPt tips only, NiPt tips, and Pt particles on the lateral surface, or Ni tips and Pt particles on the DRs.

The NiPt tips on the CdSe/CdS DRs can be used for catalytical purposes. Electrochemical measurements revealed their higher HER activity compared to that of pure Ni or Pt tips under alkaline conditions. A photocatalytic experiment, also under alkaline conditions, proves the suitability of the combination of CdSe/CdS as the light-harvesting semiconductor and NiPt as an alloyed cocatalyst for HER. The utilization of NiPt tips as cocatalysts on DRs offers great potential for optimizing activities for photocatalytic HER under alkaline conditions.

## Experimental Section

### Chemicals

All chemicals except oleylamine were used as received. Diphenylether (99%), 1,2-hexadecanediol (HDD, 90%), 11-mercaptoundecanoic acid (11-MUA, 95%), oleylamine (OAm, 70%), oleic acid (OAc, 90%), sulfur powder (S, 99.98%), and trioctylphosphine oxide (TOPO, 99%) were purchased from Sigma-Aldrich. Selenium (Se, 99.5% powder mesh 200) was purchased from Acros Organics. Hexylphosphonic acid (HPA, 99%) and octadecylphosphonic acid (ODPA, >99%) were purchased from PCI Synthesis. Trioctylphosphine (TOP, 97%) and Pt acetylacetonate (Pt­(acac)_2_, 98%) were purchased from abcr. Cadmium­(II) oxide (CdO, 99.99%) was purchased from ChemPur. 1,2-Dichlorobenzene (DCB, 98%) was purchased from Merck. Chloroform (99.8%), methanol (99.8%), and toluene (99.8%) were purchased from FisherScientific. Potassium hydroxide (KOH, >85%) was purchased from Sigma-Aldrich. Ethanol (absolute, EtOH, ≥99.8%) and Isopropanol (99.7%) were purchased from VWR. Argon (5.0) was purchased from Westfalen. Sodium hydroxide (NaOH, 99%) was purchased from Grüssing. Ni acetylacetonate (Ni­(acac)_2_) was purchased either from abcr (95%) or Thermo Scientific (96%).

For the ligand exchange, electrocatalytic, and photocatalytic measurements, ultrapure water was used in all experiments.

### Synthesis of CdSe/CdS DRs

CdSe/CdS DRs were prepared in a two-step synthesis according to a procedure of Carbone et al.^37^

Starting with the synthesis of CdSe dots, 3.00 g of TOPO, 0.280 g of ODPA (0.837 mmol), and 0.060 g of CdO (0.47 mmol) were mixed, and the reaction mixture was dried under reduced pressure at 150 °C for at least 1 h. Under a nitrogen atmosphere, the temperature was increased to 300 °C and maintained until the solution became colorless. After decolorization of the solution, 1.8 mL of TOP (4.0 mmol) was injected. The temperature was then increased to 380 °C, and 0.37 mL of a solution of selenium in TOP (2 M) (0.74 mmol) was injected. The reaction solution was cooled immediately by forced air flow, and 10 mL of toluene was added when the temperature reached 130 °C. In the first purification step, the particles were precipitated with methanol (final volume 45 mL) and centrifuged (10 min, 16,098 rcf, 20 °C). The colorless supernatant was removed in the second purification step, the colored residue was dispersed in 10 mL of toluene, and 15 mL of methanol was added. The sample was centrifuged under the conditions as before, and the procedure of the second purification step was repeated in the third purification step. The sample was then stored under inert conditions in 3 mL of TOP. The diameter and the concentration of the CdSe dots were determined by a method of Yu et al.^53^

For the encapsulation of the CdSe dots with CdS, 3.00 g of TOPO, 0.290 g of ODPA (0.867 mmol), 0.080 g of HPA (0.48 mmol), and 0.057 g of CdO (4.4 mmol) were mixed and dried under reduced pressure at 150 °C for at least 1 h. The temperature was raised to 300 °C under a nitrogen atmosphere and maintained until the solution became colorless. 1.8 mL of TOP (4.0 mmol) was injected into the colorless solution, the reaction solution was heated to 350 °C, and 1.8 mL of S:TOP (2 M) (3.6 mmol) mixed with typically 4·10^–8^ mol CdSe dots was injected. After 8 min of reaction time, the reaction solution was cooled by forced air flow, and 10 mL of toluene was injected at 130 °C. In the first purification step, the particles were precipitated with methanol (final volume 45 mL) and centrifuged (10 min, 16,098 rcf, 20 °C). In the second purification step, the colorless supernatant was removed, the colored residue was dissolved in 10 mL of toluene, and the particles were precipitated using 15 mL of methanol and centrifuged under the same conditions as in the first purification step. In the third purification step, the second purification step was repeated. The DRs were stored in 3 mL of toluene.

In order to obtain more DRs per synthesis, these were also prepared in double batch size. For this purpose, all weights and volumes were doubled, and the particles were dissolved in 3 mL of toluene after cleaning.

### Synthesis of Ni-Tipped CdSe/CdS DRs

The synthesis of Ni tips was performed by merging the protocols of Nakibli et al.^11^ and Ahrenstorf et al.^41^

In a typical synthesis, 0.0860 g of HDD (0. 332 mmol), 10 mL of diphenyl ether, 0.20 mL of OAc (0.63 mmol), and 0.050 g of Ni­(acac)_2_ (0.19 mmol) were mixed and purged with nitrogen for at least 1 h at 120 °C. A corresponding amount of methanol-precipitated and centrifuged (16,098 rcf) DRs were dissolved in 1.0 mL of TOP (2.2 mmol) under an inert atmosphere using ultrasound. In the case of a single-DR batch size, 0.500 mL of the DR solution was used, and in the case of a double-DR batch size, 0.250 mL was utilized. A total of 0.20 mL of OAm (0.61 mmol), dried and degassed via the pump-freeze–thaw procedure, was added to the reaction solution, and the temperature was increased to 200 °C. When 200 °C was reached, the DRs dissolved in TOP were injected immediately. After a reaction time of 20 min, the black solution was cooled by forced air flow, and 10 mL of toluene was added at 130 °C. In the first purification step, the particles were precipitated using isopropanol (final volume 45 mL) and centrifuged (15 min, 1197 rcf, 20 °C). The light brown supernatant was removed, and the black-brown residue was dissolved in 10 mL of toluene. Isopropanol (15 mL) was added, and the centrifugation was carried out as in the first purification step. The residue was redispersed in 1 mL of toluene.

In order to obtain more DRs with Ni tips per synthesis for subsequent experiments, the batch size of the synthesis was varied. While the volume of the diphenyl ether remained constant at 10 mL, all other weights and volumes were doubled or tripled and redispersed in 2 or 3 mL of toluene, respectively, after cleaning.

To obtain oxide-layer-free Ni tips, the particles were kept under an inert atmosphere during the cleaning process and by utilizing solvents sealed and equipped with a molecular sieve. The particles were stored under an inert atmosphere.

### Synthesis of NiPt-Tipped CdSe/CdS DRs

The synthesis of NiPt tips on DRs was carried out by performing a synthesis for Pt growth onto Ni tips, adapted from Habas et al.^34^

In a standard synthesis, 0.0430 g of HDD (0.166 mmol) and 0.10 mL of OAc (0.32 mmol) were added to 10 mL of diphenyl ether, and the solution was purged with nitrogen at 120 °C for at least 1 h. 0.500 mL of a DR sample with Ni tips was precipitated using isopropanol, centrifuged (16,098 rcf), and dissolved together with 7 mg of Pt­(acac)_2_ (0.02 mmol) in 0.50 mL of DCB. Subsequently, 0.10 mL of OAm (0.30 mmol), which was dried and degassed via the pump-freeze–thaw procedure, was added to the reaction solution, and the temperature was increased to 200 °C. The solution of DRs and Pt­(acac)_2_ was injected, and the reaction was allowed to proceed for 7 min. The reaction solution was cooled by forced air flow and diluted at 130 °C with 10 mL of toluene. The cleaning process was carried out as described for the CdSe/CdS/Ni DRs. The particles were dissolved in 0.5 mL of toluene.

In order to require smaller volumes of the CdSe/CdS/Ni samples, the batch size, including all weights and volumes, was halved in some cases.

### Ligand Exchange

The ligand exchange was accomplished by a method of Acharya et al. with slight modifications.^54^

For the phase transfer to water due to an exchange of the ligands, 55 μL of a CdSe/CdS/NiPt DR sample was diluted in 945 μL of toluene, filled up with 8 mL of methanol, and centrifuged (40 min, 16,098 rcf, 0 °C). The particles were then added to 2 mL of chloroform as well as 23.25 mg of 11-MUA and stirred for 5 min. One mL of NaOH solution (50 mg of NaOH in 14.35 mL of ultrapure water) was added, and the mixed solutions were stirred vigorously for 1 min. After phase separation, the aqueous phase was removed, and the organic phase was washed three more times with sodium hydroxide solution. Methanol was added to the combined aqueous phases (final volume 11 mL), and the mixture was centrifuged (10 min, 16,098 rcf, 20 °C). The supernatant was removed, and the residue was stored dry.

### Characterization

Transmission electron microscopy (TEM) images were acquired with a JEOL JEM 1011 at an accelerating voltage of 100 kV. High-resolution transmission electron microscopy (HRTEM) and scanning-transmission electron microscopy (STEM) were acquired at an accelerating voltage of 200 kV using a double-corrected (CESCOR and CETCOR, CEOS) JEOL JEM 2200FS microscope with an in-column image filter (Ω-type), a high-angular darkfield (HAADF) detector, and a Gatan 4K UltraScan 1000 camera. Elemental maps of energy-dispersive X-ray spectroscopy (EDX) were obtained by using a JEOL JED-2300 analyzing station with a 100 mm silicon drift detector. EELS data were obtained by recording the absorption of the primary electron beam for different energy ranges using DigitalMicrograph software with an energy resolution of 0.8 eV. The evaluation of the EELS data was carried out by using MATLAB software. For the measurements, the samples were dropped onto carbon-coated copper TEM grids with 400 meshes. ImageJ software was used to evaluate the size distribution of the different nanostructures. For each sample, a total of 200 structures were counted in different TEM images from different areas of the TEM grids where possible.

Powder X-ray diffraction (PXRD) measurements were performed using a copper anode (CuK_α_, λ = 0.154 nm) as a radiation source and a MAR345 detector of Marresearch with an integration time of 5 min. The samples were rotated by 180 ° during measurements. To prepare the samples, a glass capillary with a wall thickness of 0.01 mm and an outside diameter of 0.1 mm was dipped in the solution of the aliquots or the final sample, respectively, and the solution in the capillary was dried afterward.

The Ni K_α_ HERFD-XANES data were acquired at the ID26 beamline at the European Synchrotron Radiation Facility (ESRF) in Grenoble (France). The measurement and evaluation of the HERFD-XANES data were carried out as part of the MA5366 proposal.^55^ The intensity of the Ni K_α_ main line was measured using a Si(620) crystal in a Rowland geometry within a Johann-type X-ray emission spectrometer.^56^ The radius of crystal bending was 1 m. The monochromator and undulator gaps were scanned continuously. The beam size was 0.2 × 0.4 mm. Samples were transferred to the experiment in a self-constructed airtight cell, which had already been described in the previous work.^57^ To exclude possible radiation damage-induced changes in the spectra, a radiation damage study was performed, as described in the SI. Principally, radiation damage was avoided by collecting the HERFD-XAS spectra (acquisition time = 30 s per spectrum) on different points on the cell, and averaging 10 spectra. The normalization and linear combination of the XAS data were performed using the LARCH XAFS module.^58^

Cyclic voltammetry (CV) was performed in 0.1 M KOH (pH 13) with an Ivium Vertex.1A potentiostat using a three-electrode cell with an Ag/AgCl reference electrode, a Pt wire counter electrode, and a nanoparticle film on an FTO substrate (Ossila, TEC 15) as working electrode. For sample preparation, FTO substrates were cleaned by ultrasonification in acetone, isopropanol, and ultrapure water. The surface of the FTO substrate was covered with insulating PTFE glassfiber tape purchased from High-tech-flon, leaving only a surface area of 0.07 cm^2^ uncovered. Onto the free FTO surface, 2 μL of 5 mg/mL dispersions of nanoparticles in toluene was dropcasted. The electrolyte was degassed with nitrogen for 30 min prior to the start of the measurements, and the cell was flushed with nitrogen throughout the measurements. To test the catalytic activity in the HER, CV was performed up to a cathodic switching potential of −0.55 V vs RHE.

The photocatalytic experiments were performed according to a procedure of Hentschel et al. with slight modifications.^33^ The experiments were carried out in a flow-through system that was constantly fed with 10 sccm of argon. A partially filled, gastight quartz cuvette (1.0 cm path length, 3.0 mL liquid sample, and 0.5 mL gas volume) was integrated into this system. The carrier gas flow was set by using mass flow controllers (Alicat Scientific, 10 sccm, 2 barA front pressure, and atmospheric back pressure), and the temperature of the liquid in the quartz cuvette was kept at 293 K using a Peltier element (3.0 cm × 1.2 cm) connected to a TEC controller (Meerstetter, TEC-1089-SV). The system was purged with argon at the beginning of the experiment for 2 h and 35 min at 1500 rpm stirring until the O_2_ content reached a constant value. The gas composition was monitored at all times by using a quadrupole mass spectrometer (Hiden Analytical, HPR-20 EGA, 50 amu mass range, QGA 2). After the purging-process completion, the photocatalytic experiment was started by turning a 365 nm LED on (Thorlabs, SOLIS-365C). The collimated LED light beam (constant current = 765 mA) was limited to a cross-section of 1.5 cm^2^ using 3D-printed apertures. The resulting power densities of 65 mW/cm^2^ were quantified by using a thermal power sensor (Thorlabs, S425C). For sample preparation, the 11-MUA-capped NiPt-tipped DRs were dissolved in 5 M NaOH (pH 14.7) and 10% EtOH as a hole scavenger using ultrasonication to obtain 3.0 mL liquid sample. The optical density of the liquid sample was determined at 0.4 using an absorption spectrometer (Cary5000, Agilent) equipped with an external integrating sphere.

## Supplementary Material



## Notes

Details on structure of CdSe/CdS DRs, optical properties of bare, Ni-tipped, and NiPt-tipped CdSe/CdS DRs, assignment of lattice planes, compositional analysis using EDX, changing the Pt-to-Ni ratio, NiPt tip size, HERFD-XANES data, EELS data, additional data for oxide-layer conversion, and additional data regarding catalysis (PDF)
