# Supplementary material for: Seed-Mediated Synthesis of NiPt-Alloy-Tipped CdSe/CdS Nanocrystals for Photocatalysis
Source: Chem Mater. 2026 Mar 19;38(7):3338–49. doi: 10.1021/acs.chemmater.5c03047 (PMC13084996; doi:10.1021/acs.chemmater.5c03047)
Supplement: Supplementary file 1 [file cm5c03047_si_001.pdf]

Supporting Information for

Seed-Mediated Synthesis of NiPt-Alloy-Tipped CdSe/CdS Nanocrystals for Photocatalysis

Mareike Dittmar,† Julia Voß,† Sebastian Hentschel,† Lars Klemeyer,‡ Dorota Koziej,‡ Dennis Bonatz,† Charlotte Ruhmlieb,† Tobias Kipp,*,† and Alf Mews†

†*University of Hamburg, Institute of Physical Chemistry, Grindelallee 117, 20146 Hamburg, Germany*

‡*University of Hamburg, Institute for Nanostructure and Solid-State Physics, Luruper Chaussee 149, 22761 Hamburg, Germany*

E-mail: tobias.kipp@uni-hamburg.de

Phone: +49 40 2395-28277

Structure of CdSe/CdS DRs

Figure S1a shows a representative TEM image of the CdSe/CdS DRs used as precursor structures for the synthesis of Ni-tipped DRs. The DRs were prepared with CdSe dots with a diameter of 2.3 nm. Figure S1b shows a XRD pattern of the CdSe/CdS DRs. The pattern is dominated by the reflections originating from the CdS shell.

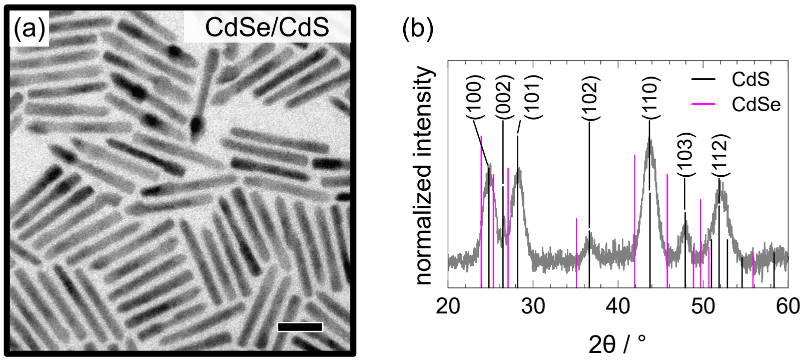

Figure S1: (a) Representative TEM image and (b) PXRD pattern of the CdSe/CdS DRs. Reference data for CdS (PDF#: 00-041-1049), with marks for the dominant reflections, and for CdSe (PDF#: 00-008-0459) are given as well.

Optical properties of bare, Ni-tipped, and NiPt-tipped CdSe/CdS DRs

Figure S2 shows (a) UV-vis absorption spectra, (b) fluorescence spectra, and (c) fluorescence decay curves of the bare DRs (black), the Ni-tipped DRs (blue), and the NiPt-tipped DRs (red). The prominent feature in the absorption spectra is the excitonic transitions within the CdS shell for wavelengths $<$ 500 nm. The absorption of the CdSe shell can be seen at about 550 nm. In the case of metal-tipped DRs, these features are superimposed by a broad absorbance over the whole spectral range, increasing from long to shorter wavelengths. The fluorescence spectra reveal nearly no influence on the emission wavelength by the metal tips. The fluorescence decay, however, is drastically shortened by both, the Ni and NiPt tips. The metal tips induce strong nonradiative processes, like charge and energy transfer, which drastically reduce the photoluminescence quantum yield (QY). The QYs have been

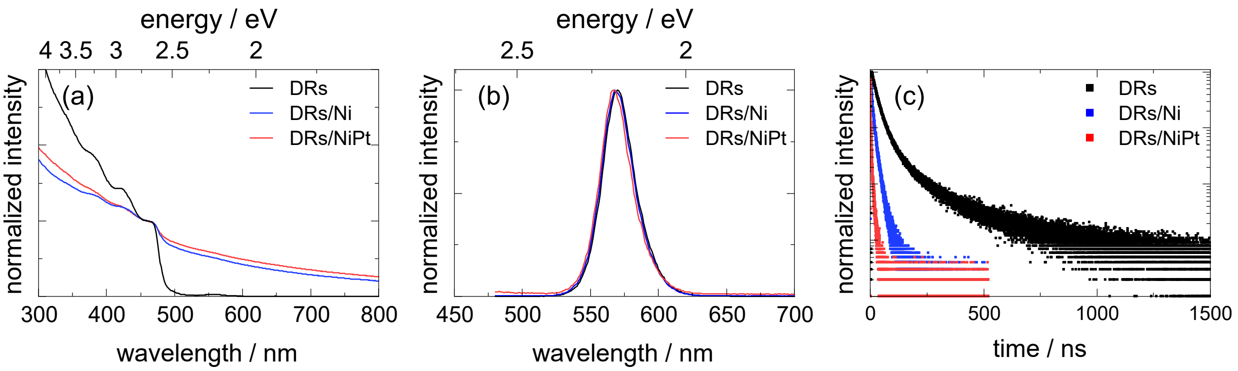

Figure S2: Optical properties of bare (black), Ni-tipped (blue), and NiPt-tipped (red) DRs. (a) UV-vis absorption spectra normalized to the first excitonic transition within the CdS shell at a wavelength of 460 nm. (b) Normalized fluorescence spectra. (c) Normalized fluorescence decay curves on a log scale.

determined relative to Rhodamin 6G to be 34% for the bare DRs, 1.5% for Ni-tipped DRs and below 1% for NiPt-tipped DRs.

The UV-vis absorbance spectra were obtained on samples diluted in toluene using a Cary 5000 UV-vis-NIR spectrophotometer from Varian in single-beam mode. The PL spectra were recorded using a FluoroMax-4 spectrofluorometer from Horiba Jobin Yvon. A PicoQuant Fluorotime 300 photoluminescence spectrometer was used to record the fluorescence decay curves. Here, the samples were excited with an NKT SuperK Fianium white light laser set at a wavelength of 460 nm. The repetition rate was set to 1.89 MHz for metal-tipped DRs or 0.590 MHz for the DRs without metal tips. The number of detected photons per excitation pulse (pile-up rate) was kept below 1 %. The measurement was stopped at an intensity of 10 000 events.

Assignment of lattice planes

The lattice planes of the NiPt tips were assigned by determining the lattice spacings using Fourier transformations of HRTEM images. The spacing of the lattice planes visible in Fig. 1d of the main text was determined to be 1.90 Å, which is between the d-spacings of the (200) lattice planes in Ni and Pt (1.76 Å and 1.96 Å, respectively). In Fig. 1e of the main text, the spacing of the lattice planes is 2.25 Å, which is between the (111) lattice spacings of Ni and

Pt (2.03 Å and 2.27 Å, respectively). The assignment of the lattice planes is unambiguous since higher-indexed planes have smaller spacings. Overall, HRTEM images of 20 different tips were analyzed according to this method. Most often, the (111) and the (200) lattice planes were visible.

Compositional analysis using EDX

Energy dispersive X-ray spectroscopy (EDX) was employed to analyze the average Pt-to-Ni ratio within the NiPt-tipped DR sample. Figure S3 shows the field of view (approx. 885 nm in diameter) of a TEM measurement, in which a large number (> 700) of tips have been imaged. The sum EDX spectrum obtained from the whole imaged area was analyzed. To quantify the Pt and Ni content, the Pt L_α and the Ni K_α signals have been used. Here, the Pt-to-Ni ratio was determined to be 51:49. Analyzing further TEM images such that overall far more than 1000 tips are investigated, an average ratio of 50:50 has been found.

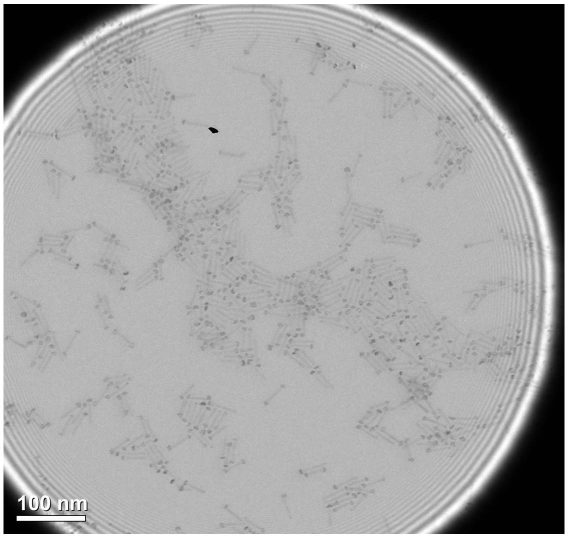

Figure S3: TEM field of view, imaging a large number of NiPt-tipped DRs. The Pt and Ni content of this sample has been analyzed using the EDX sum spectrum.

Changing the Pt-to-Ni ratio

The Pt content of the NiPt tips attached to the DRs can be altered by adjusting the Pt content of the precursor solution. Figure S4 shows the platinum content of DRs with NiPt tips, as determined by XRD using Vegard's law, in relation to the amount of platinum precursor used. The relative platinum content could be adjusted to between around 45 and 70 %. Note that we used a different batch of Ni-tipped DRs than the one shown in Fig. 1a in the main text. The Ni-tipped DRs used had a tip size of 7.0 ± 0.8 nm; for about 78 % of all structures, both apexes were decorated with Ni, 19 % exhibited only one tip.

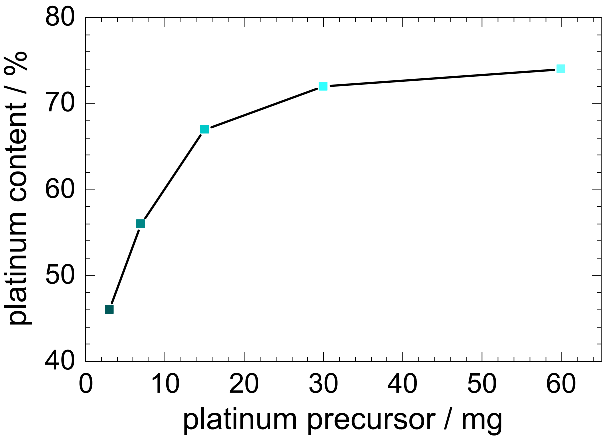

Figure S4: Platinum content of the NiPt tip *vs.* platinum-precursor amount.

NiPt tip size

The sizes of the metal tips of different samples were determined *via* TEM, by measuring the tip diameter at its thickest cross-section perpendicular to the nanorod axis. Figure S5a shows the data set obtained for Ni tips handled under ambient conditions. Exemplary corresponding TEM images are shown in Fig. 1 and Fig. 2 of the main text. It can be seen that the tip diameter is nearly unchanged within the first minutes of the reaction and is slightly increasing after $t_r = 5\,\mathrm{min}$.

Figure S5b shows the data set obtained for Ni tips handled under inert conditions. Ex-

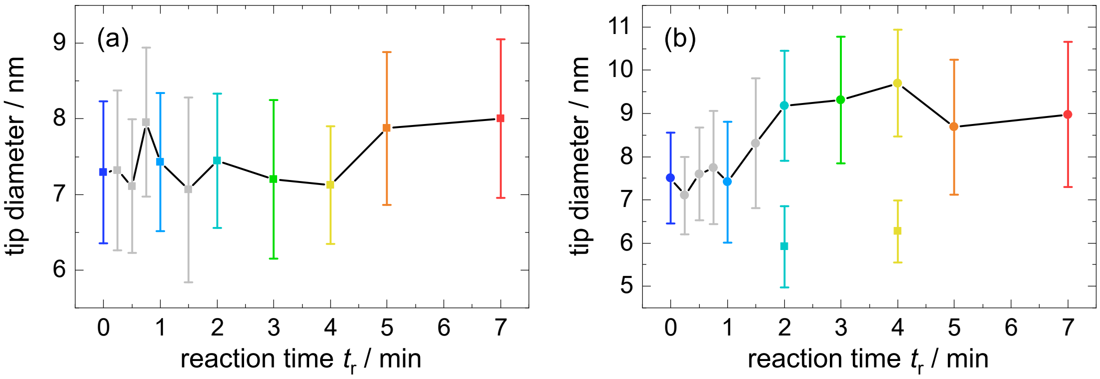

Figure S5: Tip diameter of the aliquots of the NiPt formation with Ni tips handled under (a) ambient and (b) inert conditions. The color scheme corresponds to the color scheme of the data in the main document (cf. Fig. 1, Fig. 2. and Fig. 4). Light gray data points correspond to samples, from which the TEM data are not shown due to redundancy.

emplary corresponding TEM images are given in Fig. 5 of the main text. Here, the tip diameter starts to increase after $t_r = 1.5\,\text{min}$.

HERFD-XANES data

In the main text it was elucidated that the HERFD-XANES spectrum of the sample exposed to air can be well reproduced by superimposing the spectra of the Ni-tipped DR samples stored under nitrogen and the NiO-reference spectrum with weighting factors of 0.66 and 0.34, respectively.

For a further analysis of the HERFD-XANES data, Fig. S6 shows the difference spectrum of the sample exposed to air and that of the sample stored under nitrogen, weighted by a factor of 0.66. For a perfect fit, this difference spectrum represents signal from the material that is formed due to air exposure. For comparison, Fig. S6 also shows the NiO powder reference spectrum (measured in HERFD-XANES and transmission mode) and NiOH powder reference spectrum.

The difference spectrum shown in Fig. S6 exhibit characteristic spectral features that can be matched with NiO references. The position of the pre-edge feature (I) aligns well with the NiO pre-edge (black). Furthermore, the difference spectrum displays a shoulder

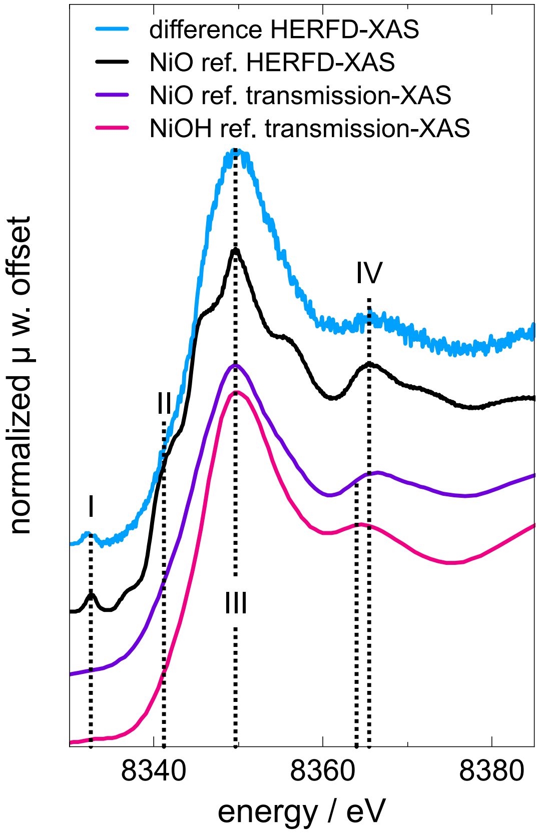

Figure S6: Comparison of the difference spectra of the sample exposed to air and that of the sample stored under nitrogen, weighted by a factor of 0.66 (light blue) with HERFD-XAS of NiO (black) and transmission XAS reference of powder NiO (purple) and powder NiOH spectra (pink) used with permission from the X-ray Absorption Data Library (University of Chicago).[1,2]

(II) in the white line (III), which is characteristic of NiO spectra. While the energy position of the white line (III) is similar for NiO and NiOH, the maximum of the post-edge feature (IV) differs between NiO and NiOH by approximately 1.5 eV. The maximum of the spectral feature (IV) is located at 8365.5 eV, consistent with the NiO references. Similar shifts of approximately 1.5 eV in the post-edge peaks (feature IV) have been reported for NiOOH in the literature.[3] Therefore, we conclude that a NiO contribution dominates the difference spectra.

Remaining slight deviations between the difference spectrum and the NiO HERFD-XAS reference may arise from differences in domain size (macroscopic foil vs. nanomaterials) and from self-absorption effects. In addition, the transmission XAS references of NiO (purple)

and NiOH (pink) have limited resolution due to the measurement geometry and a step size of 0.5 eV, which explains why pre-edge features are barely resolved.

Figure S7 shows results of a radiation-damage study for Ni-tipped DRs that were stored under nitrogen atmosphere (panel (a)) and that were exposed to air (panel (b)). X-ray absorption spectra were recorded at various times after X-ray exposure. The spectra did not change significantly over a 240-second period, proving that sample degradation does not influence our data interpretation. Thus, we chose 30 s for the acquisition time and averaged over 10 spectra for better signal to noise statistic, as explained in the experimental section.

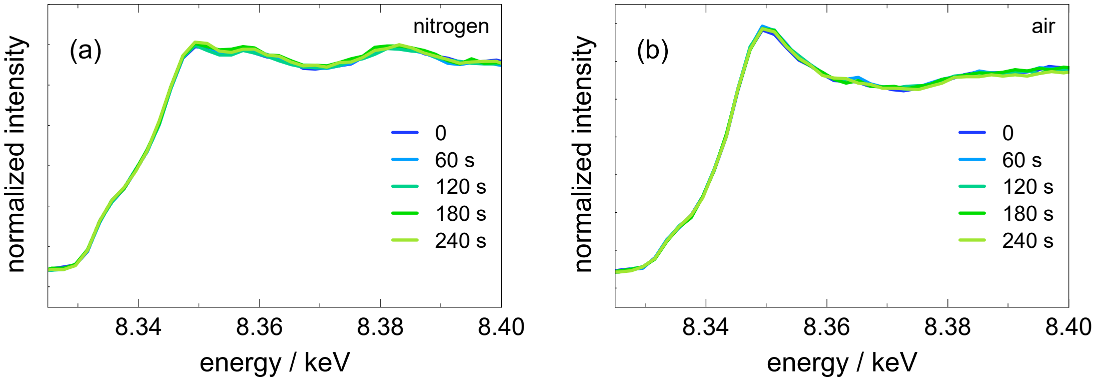

Figure S7: XAS data obtained for Ni-tipped DRs that were either (a) stored under nitrogen or (b) exposed to air. The spectra were taken after 0 s, 60 s, 120 s, 180 s, and 240 s X-ray exposure time. The acquisition time of each spectrum was 60 s.

EELS data

Figure S8 depicts the EELS data of aliquots belonging to reaction times $t_r = 3\,\text{min}$ and $t_r = 7\,\text{min}$ (green and blue curves, respectively) together with reference spectra for Ni and NiO. Figure S8a shows the L-edge of Ni, while Figure S8b shows the K-edge of oxygen. Our experimental spectra were baseline corrected following a method of Fung and coworkers.[4] Furthermore, they were shifted in energy by a few eV, due to offsets compared to the reference spectra, possibly resulting from charging effects. In the following discussion, we will compare only peak widths and intensities.

Figure S8a reveals that the shape of the spectrum for $t_r = 7\,\text{min}$ is very similar to the

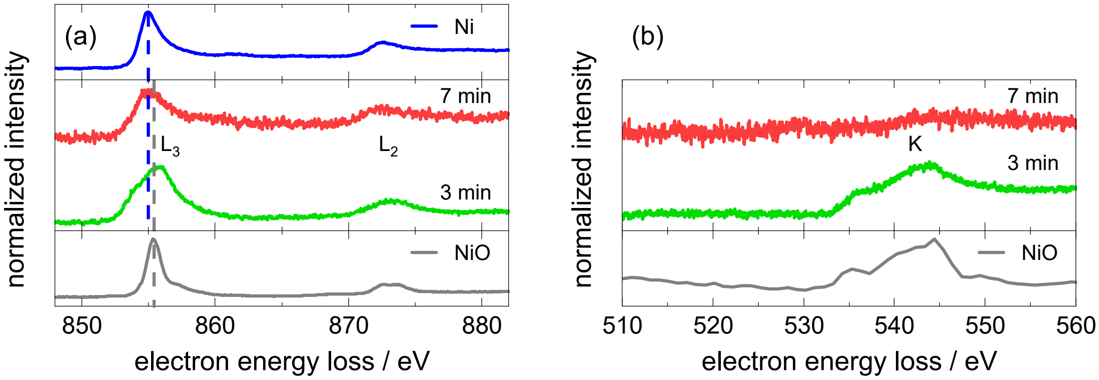

Figure S8: (a) Ni L-edges and (b) oxygen K-edges of the EELS spectra of the CdSe/CdS/NiPt DRs after background subtraction after 3 min and 7 min reaction time. The Ni and NiO references in (a) are from Potapov *et al.*[5] and were downloaded from the EELS database.[6] The NiO references were obtained from the EELS atlas.[7] All spectra were normalized to the intensity maximum and vertically shifted for clarity.

Ni-reference spectrum, while the spectrum for t_r = 3 min exhibits broader peaks. Here, the line shape of the L_3-edge peak can be described by a superposition of two overlapping peaks with the higher-energy one being more pronounced than the lower-energy one. Since the reference NiO peak is shifted to higher energies compared to the reference Ni signal, this suggests that for t_r = 3 min the sample contains a considerable amount of NiO, which is not existent for the sample belonging to t_r = 7 min. The fundamentally different NiO content in both samples can also be deduced from their EELS spectra at the oxygen K-edge, as shown in Fig. S8b. The spectrum from the aliquot belonging to t_r = 3 min exhibits a pronounced K-edge signal, similar to the NiO reference peak, while for t_r = 7 this signal is nearly completely gone.

EELS data also allows for the analysis of the oxidation state of the Ni. Following a work of Graetz et al.,[8] the edge fine structure, in particular the L_3/L_2 ratio (white line ratio), is dependent on the oxidation state. To calculate the white line ratios, the L-peak intensities corrected for a linearly fitted background, were integrated over 10 eV broad intervals. The white line ratios were calculated by integrating the L-peak intensities, corrected for linearly fitted backgrounds, over 10 eV intervals. Results are given in Table S1. The ratio calculated for the aliquot belonging to t_r = 3 min is between those of the references for Ni and NiO,

indicating the presence of both elemental Ni and NiO. For $t_r = 7\,\mathrm{min}$, a value close to the Ni reference is found, confirming the conversion of the NiO layer.

Table S1: White line ratios L_3/L_2 for the references from Potapov *et al.*[5] and the analyzed aliquot or sample.

sample	Ni L_3/L_2 ratio
NiO reference	4.17
3 min	3.78
7 min	3.10
Ni reference	3.33

Additional data for oxide-layer conversion

Figure S9 shows TEM images of the DRs with Ni tips utilized as precursors for the particles presented in Fig. 7 of the main document. Figure S9a depicts the Ni-tipped DRs used for the synthesis of the samples shown in Fig. 7a-b and 7e-f. Figure S9b depicts the Ni-tipped DRs utilized for the samples shown in Fig. 7c and 7d. For the samples with air contact, the DRs with Ni tips that had been produced, processed and stored under inert conditions were allowed to oxidize for one to two days after air contact until NiPt-synthesis. Figure S9c shows the Ni-tipped DRs employed in the synthesis of the samples shown in Fig. 7g and 7h. The Ni-tipped DRs shown in Fig. S9a and S9c were processed under ambient conditions while the ones of Fig. S9b were initially processed under inert conditions. However, it has to be noted that all samples were in contact with air for different durations before the TEM images were taken.

Figure S10 compares PXRD data measured for the samples shown in Fig. 7a-d with literature data of Ni, Pt, and CdS. For the samples shown in Fig. 7a and 7b, for which the Ni-tipped DR precursors were handled either under ambient or inert conditions, and for which OAc, OAm, and HDD was used in the synthesis, a NiPt reflection is present (red and orange curves in Fig. S10). For the sample shown in Fig. 7c, for which OAc was omitted, only a pronounced Pt reflection is observable (yellow curve in Fig. S10). For the sample

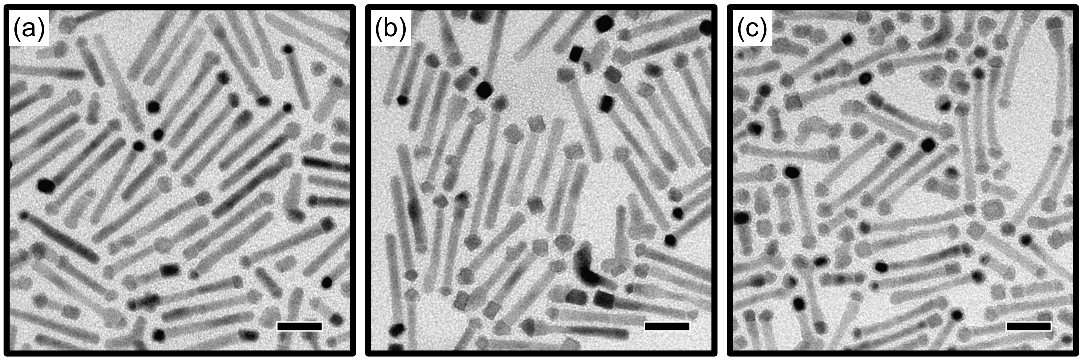

Figure S9: TEM images of the CdSe/CdS/Ni DRs used for the particles shown in Fig. 7 of the main text. (Scale bar 20 nm).

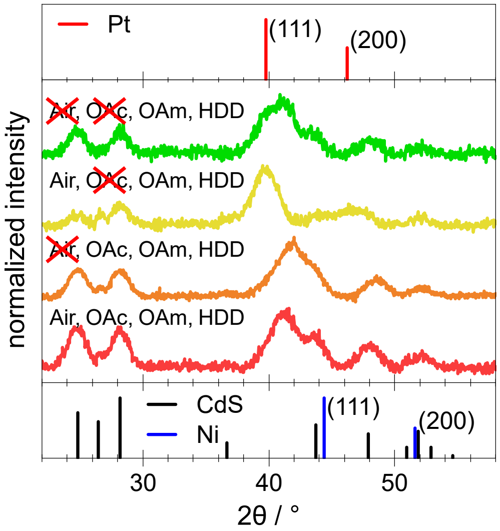

Figure S10: PXRD data measured for the samples shown in Fig. 7a (red), Fig. 7b (orange), Fig. 7c (yellow), and Fig. 7d (green) of the main text. Top and bottom subpanels give literature data for Pt (PDF#: 00-004-0802), Ni (PDF#: 00-004-0850), and CdS (PDF#: 00-041-1049).

shown in Fig. 7d, for which OAc was omitted and the Ni-tipped DRs were handled under inert conditions, both a NiPt and a Pt reflection are recognizable in close proximity (green line in Fig. S10). These findings perfectly support the ones deduced in the course of the discussion of Fig. 7a-d of the main text.

Additional data regarding catalysis

For the electrochemical measurements shown in Fig. 7b of the main text, the utilized DRs had an average length of 44 ± 4 nm and diameter of 5.5 ± 0.5 nm. Onto these DRs, metal tips were grown.

The preparation of the Ni-tipped and NiPt-tipped DRs was carried out by the presented methods. The Ni tips had a bimodal size distribution with maxima at 9 ± 2 nm and 14 ± 1 nm. Around 5 % of the DRs were tipless, while around 15 % of particles had two tips. The NiPt tips had an average diameter of 13 ± 1 nm. About 15 % of the DRs in the NiPt-tipped sample were tipless, around 20 % had two tips.

The synthesis of Pt tips was performed by a method of Habas and coworkers.[9] The Pt-tip diameter was broadly distributed between 1 and 7 nm. About 30 % of the DRs were tipless, while around 20 % had a secondary tip. 2 % of DRs even had more than two metal particles on their surface.

Figure S11a shows a representative TEM image of the NiPt-tipped DR samples before solvent change. Evaluating approximately 200 particles, we find that about 75 % of the particles had exactly one tip, around 20 % had two tips and only 1.5 % without metal tips were present. Figure S11b shows a representative TEM image of the NiPt-tipped DR sample taken from the alkaline photocatalysis solution before the actual photocatalysis experiment. This sample has been cleaned two times with ultrapure water under centrifugation for the TEM investigation. Evaluating 66 particles, we find that half of the particles had lost their tip. NiPt particles themselves can absorb (or scatter) light, even when they are not attached to a semiconductor and therefore do not contribute to the HER as a cocatalyst. The apparent QY is sensitive to the light absorbed/scattered and big metal tips can reduce the apparent QY by absorbing or scattering photons, which is difficult to quantify during HER. Figure S11c shows the absorbance spectra of the DRs and the corresponding NiPt-tipped DRs, as well as difference spectra of the NiPt-tipped DRs spectrum and DR spectra multiplied by different factors. The difference spectra imitate the spectrum of the pure NiPt particles.

This factor provides information about the light absorbed by the semiconductor and the metal. In this case, a factor of 0.25 produces a nearly exponential curve, in line with the expected metal absorbance. The difference at 365 nm is marked with a red arrow and shows that only around 25 % of the light is absorbed by the semiconductor.

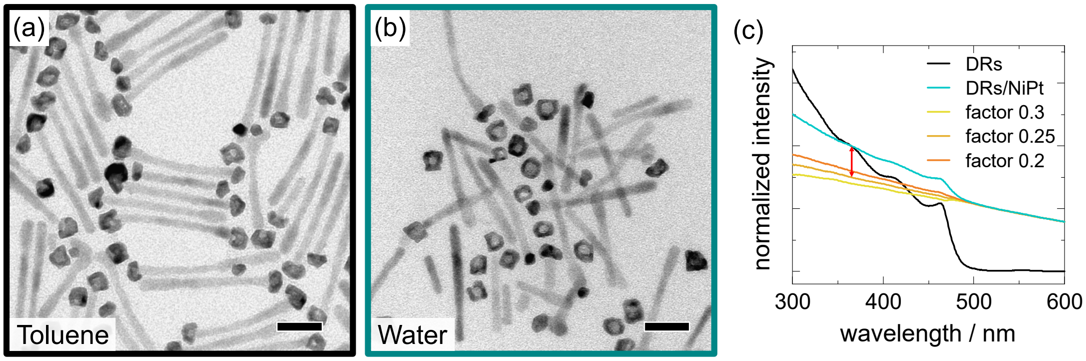

Figure S11: TEM images of the DR sample with NiPt tips (a) before in toluene and (b) after the ligand exchange, dissolving them in the catalysis medium (5 M NaOH/10 % EtOH) and cleaning them for TEM. (c) Absorbance spectra of DRs and NiPt-tipped DRs normalized to the intensity at 365 nm. From the spectrum of the NiPt-tipped DRs, the absorbance of bare DRs, multiplied with a factor (0.2, 0.25 and 0.3), was subtracted. The absorbance difference at 365 nm is marked with a red arrow.

References

(1) XAS Database (XASDB), Canadian Light Source, available at: http://xasdb.lightsource.ca, sample ID: nclzvt (NiO).

(2) XAS Database (XASDB), Canadian Light Source, available at: http://xasdb.lightsource.ca, sample ID: hcyn08 (NiOH).

(3) Mao, X.; Chang, C. W.; Li, Z.; Han, Z.; Gao, J.; Lyons, M.; Sterbinsky, G.; Guo, Y.; Zhang, B.; Wang, Y.; Wang, X.; Han, D.; Yang, Q. H.; Feng, Z.; Weng, Z. Sustainedly High-Rate Electroreduction of CO2 to Multi-Carbon Products on Nickel Oxygenate/Copper Interfacial Catalysts. *Advanced Energy Materials* **2024**, *14*, 1–8, DOI: 10.1002/aenm.202400827.

(4) Fung, K. L.; Fay, M. W.; Collins, S. M.; Kepaptsoglou, D. M.; Skowron, S. T.; Ramasse, Q. M.; Khlobystov, A. N. Accurate EELS background subtraction – an adaptable method in MATLAB. *Ultramicroscopy* **2020**, *217*, DOI: 10.1016/j.ultramic.2020.113052.

(5) Potapov, P. L.; Kulkova, S. E.; Schryvers, D.; Verbeeck, J. Structural and chemical effects on EELS L3,2 ionization edges in Ni-based intermetallic compounds. *Physical Review B - Condensed Matter and Materials Physics* **2001**, *64*, 1841101–1841109, DOI: 10.1103/physrevb.64.184110.

(6) Ewels, P.; Sikora, T.; Serin, V.; Ewels, C. P.; Lajaunie, L. A Complete Overhaul of the Electron Energy-Loss Spectroscopy and X-Ray Absorption Spectroscopy Database: eelsdb.eu. *Microscopy and Microanalysis* **2016**, *22*, 717–724, DOI: 10.1017/S1431927616000179.

(7) EELS Atlas, URL: https://eels.info/atlasetzter (access 10.05.2024).

(8) Graetz, J.; Ahn, C. C.; Ouyang, H.; Rez, P.; Fultz, B. White lines and d-band occupancy for the 3d transition-metal oxides and lithium transition-metal oxides. *Physical Review B - Condensed Matter and Materials Physics* **2004**, *69*, 235103, DOI: 10.1103/PhysRevB.69.235103.

(9) Habas, S. E.; Yang, P.; Mokari, T. Selective Growth of Metal and Binary Metal Tips on CdS Nanorods. *Journal of the American Chemical Society* **2008**, *130*, 3294–3295, DOI: 10.1021/ja800104w.
